# Integrative Ligand-Based Pharmacophore Modeling, Virtual Screening, and Molecular Docking Simulation Approaches Identified Potential Lead Compounds against Pancreatic Cancer by Targeting FAK1

**DOI:** 10.3390/ph16010120

**Published:** 2023-01-13

**Authors:** Mohammad Habibur Rahman Molla, Mohammed Othman Aljahdali, Md Afsar Ahmed Sumon, Amer H. Asseri, Hisham N. Altayb, Md. Shafiqul Islam, Ahad Amer Alsaiari, F. A. Dain Md Opo, Nushrat Jahan, Foysal Ahammad, Farhan Mohammad

**Affiliations:** 1 Department of Biological Sciences, Faculty of Science, King Abdulaziz University, Jeddah 21598, Saudi Arabia; moaljahdali@kau.edu.sa (M.O.A.); md.opu1990@gmail.com (F.A.D.M.O.); 2 Brahmanbaria Ornamental Fish Breeding and Research Centre, Brahmanbaria 3400, Bangladesh; 3 Marine Biology Department, Faculty of Marine Sciences, King Abdulaziz University, Jeddah 21598, Saudi Arabia; afsar.sumon@gmail.com; 4 Department of Biochemistry, Faculty of Sciences, King Abdulaziz University, Jeddah 21589, Saudi Arabia; ahasseri@kau.edu.sa (A.H.A.); hdemmahom@kau.edu.sa (H.N.A.); 5 Centre for Artificial Intelligence in Precision Medicine, King Abdulaziz University, Jeddah 21589, Saudi Arabia; 6 Institute of Marine Sciences, University of Chittagong, Chittagong 4431, Bangladesh; prof.shafiqimsfcu@gmail.com; 7 Clinical Laboratories Science Department, College of Applied Medical Science, Taif University, Taif 21944, Saudi Arabia; ahadamer@tu.edu.sa; 8 Department of Animal Husbandry, Patuakhali Science and Technology University, Dumki-Patuakhali Highway 8602, Bangladesh; nushratmaliha13@gmail.com; 9 Division of Biological and Biomedical Sciences (BBS), College of Health and Life Sciences (CHLS), Hamad Bin Khalifa University (HBKU), Doha 122104, Qatar; foysalgebt@gmail.com

**Keywords:** pancreatic cancer, FAK1 protein, ligand-based pharmacophore drug design, purchasable compounds, molecular docking, ADMET, MD simulation, MM-GBSA

## Abstract

Pancreatic cancer is a very deadly disease with a 5-year survival rate, making it one of the leading causes of cancer-related deaths globally. Focal adhesion kinase 1 (FAK1) is a ubiquitously expressed protein in pancreatic cancer. FAK, a tyrosine kinase that is overexpressed in cancer cells, is crucial for the development of tumors into malignant phenotypes. FAK functions in response to extracellular signals by triggering transmembrane receptor signaling, which enhances focal adhesion turnover, cell adhesion, cell migration, and gene expression. The ligand-based drug design approach was used to identify potential compounds against the target protein, which included molecular docking: ADME (absorption, distribution, metabolism, and excretion), toxicity, molecular dynamics (MD) simulation, and molecular mechanics generalized born surface area (MM-GBSA). Following the retrieval of twenty hits, four compounds were selected for further evaluation based on a molecular docking approach. Three newly discovered compounds, including PubChem CID24601203, CID1893370, and CID16355541, with binding scores of −10.4, −10.1, and −9.7 kcal/mol, respectively, may serve as lead compounds for the treatment of pancreatic cancer associated with FAK1. The ADME (absorption, distribution, metabolism, and excretion) and toxicity analyses demonstrated that the compounds were effective and nontoxic. However, further wet laboratory investigations are required to evaluate the activity of the drugs against the cancer.

## 1. Introduction

Pancreatic cancer is a disease that occurs when cells divide uncontrollably and spread into nearby tissues. Currently, it is considered to be the seventh leading cause of cancer-related deaths in the world [1]. However, PANC presents five highly alarming features in addition to a very poor prognosis [2]. The mortality rate of PANC is almost equal to its incidence rate since 458,918 new cases and 432,242 deaths were recorded in 2018 for the disease [1]. Consequently, the overall 5-year survival rate for PANC is approximately 6%, making it the most lethal cancer of them all. Furthermore, it is characterized by drug-resistance development, making it difficult to treat. The genetic component of PANC is also very complex because the genes responsible for PANC predisposition are not well-understood [3]. However, several genes have been implicated in PANC development and progression [4]. As a result, the drugs currently used to treat PANC are limited in their effectiveness since they primarily act as adjuvants and have specific mechanisms of action [5]. Considering these aspects, new and more effective anti-PANC chemotherapeutics are urgently needed. New compounds that are capable of functioning as multi-target medicines and inhibiting several PANC-related proteins alongside should be identified [6].

Moreover, focal adhesion kinase 1 (FAK1) is a ubiquitously expressed protein in pancreatic cancer; nevertheless, its expression in hematopoietic cell lineages is limited [7]. FAK is commonly overexpressed and activated in a variety of cancers and plays an important role as a targetable kinase in cancer therapy [8]. There are three main domains within FAK: the N-terminal band; ezrin, radixin, moesin homology (FERM), central kinase, and C-terminal focal adhesion targeting (FAT) [9]. It was once thought that FAK was mainly localized to the cytosol and plasma membrane since it is a key mediator of integrin signaling through its association with focal adhesion proteins, such as talin and paxillin [10]. However, the functions of FAK can be categorized into two main categories: cytosolic and nuclear [11]. FAK functions in response to extracellular signals by triggering transmembrane receptor signaling, which enhances focal adhesion turnover, cell adhesion, cell migration, and gene expression, and lead to cancer cell proliferation, survival and chemoresistance [12]. Furthermore, in advanced level of cancers, FAK is activated and/or overexpressed, promoting cancer progression and metastasis. FAK’s cytosolic signaling activities in cancer cells are highly dependent on its activity [13]. The overexpression of FAK resulting from gene amplification or mRNA upregulation in advanced human cancers is often associated with FAK activation, contributing to poor prognosis [7]. A new layer of complexity has been added to FAK signaling through the discovery of nuclear FAK regulation of gene expression. One of the first kinase-independent scaffolding functions of FAK in the nucleus was discovered to be its ability to stabilize a p53-MDM2 [14]. It has been demonstrated that FAK regulate each other to generate aggressive tumors in several studies [15].

Computer-aided drug design (CADD) is a very useful tool for various therapeutic technique to minimize the time for identification, characterization and structure-optimization [16]. It can also be useful for the rational design of prodrugs that are typically designed to increase the specificity or bioavailability of the original drug molecules [17]. However, the ligand-based approach to drug development involves studying molecules that interact with biological targets of interest to develop pharmacologically active compounds. The ideal compounds against a specific target can be selected based on a molecular-docking-based scoring function, and interaction can be documented through the analysis of the different docking poses [18]. The purpose of this study was to further our understanding of the interactions involved in the inhibition of the various proteins listed in the Protein Data Bank (PDB) by using the molecular docking approach [19]. The ADMET properties of compounds, which indicate their efficacy and toxicity, can be easily predicted using computer-aided methods, where molecular dynamic simulation confirms a drug candidate’s stability for the targeted protein. Molecular mechanics with generalized born and surface area solvation (MM/GBSA) confirmed the estimation of free energy from the protein–ligand interaction. Furthermore, a ligand-based design method may be useful when 3D structures are not available for experiments. Nevertheless, due to the lack of an experimental structure, the known ligand molecules that bind to the drug target are studied to understand their structure and physicochemical properties, with a correlation to their pharmacological activity. Therefore, ligand-based methods may utilize natural products or substrate analogs that interact with the target molecule, resulting in desired pharmacological effects through the CADD approaches, including molecular docking, ADMET, MD simulation and MM-GBSA methods.

## 2. Results

### 2.1. Ligand-Based Pharmacophore Modeling and Virtual Screening

#### Pharmacophore Model Generation

Focal adhesion kinase 1 (FAK1) is a non-receptor tyrosine kinase with key roles in the regulation of cell adhesion migration, proliferation and survival. In cancer, FAK is a major driver of invasion and metastasis, and its upregulation is associated with poor patient prognosis. Over-expression of the protein is responsible for developing pancreatic cancers. Therefore, purchasable compounds identical to the previously originated antagonist can be developed as a drug instead of a chemically synthesized compound. Twenty (20) chemically synthesis active antagonist of FAK1 (Table 1) were collected through ChEMBL and advanced literature search, which were docked with FAK1 protein. The best binding score found for the antagonist CHEMBL3657364 (PubChem CID: 58522531) was − 9.0 kcal/mol; the binding energy of other 9 molecules is shown in Table 1. Additionally, the interaction between FAK1 protein and antagonist is provided in Table S1.

Drug design requires the determination of a protein 3D structure, and nowadays the most validated structure can be obtained from several protein data banks or by homology modeling. A crystal structure of FAK1 (PDB: 3BZ3) in a complex with a compound was determined, and a ligand-based pharmacophore model of the enzymatic cavity was developed. The experimentally determined affinity of the selected ligand for FAK1 protein was validated by X-ray diffraction with an IC50 value. As a result of binding the inhibitor to FAK1, the overall expression can be regulated. The improper binding of inhibitors can sometimes result in poor efficacy against any given protein. In order for active series of inhibitors to be determined, they must have sufficient interaction to produce significantly more biological activity compared with the existing inhibitor series. The ligand-based pharmacophore model was used to generate key chemical features using Ligand Scout4.3 essential molecular design software. The ten-ligand based model was established, and model 1 was chosen based on the best score (0.9180) (Table S2).

**Figure 1:**
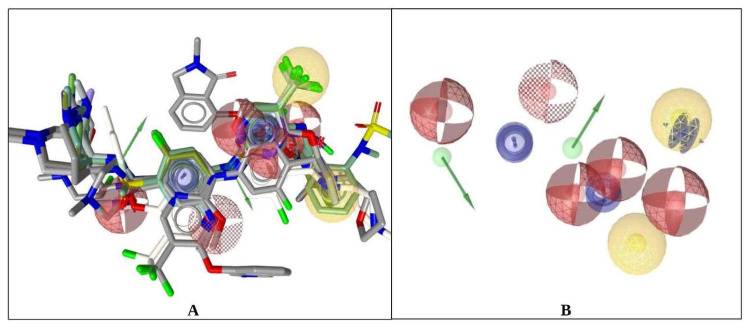
The 3D structure-based ligand-based model of FAK1 protein. (**A**) Ligand-based pharmacophore features (Model 1) were generated after complex interaction. (**B**) Two yellow spherical shapes indicating hydrophobic interaction, one blue star shape depicting the positive ionizable with tolerance, five red colors spherical shapes indicating H bond acceptor, two hydrogen bond donors represented by green spherical, or arrow shape are identified within the protein–ligand complex interaction.

The different chemical features were determined, and the total number was 10. Among them, two hydrophobics, three aromatic ring bonds, five H bond acceptors, two H bond donors, and a few exclusion volume features were presented as a protein–ligand complex interaction (Figure 1). To maintain the optimal ligand-based pharmacophore features, some features were omitted during the time of pharmacophore model generation.

### 2.2. Pharmacophore Model Validation

**Figure 2:**
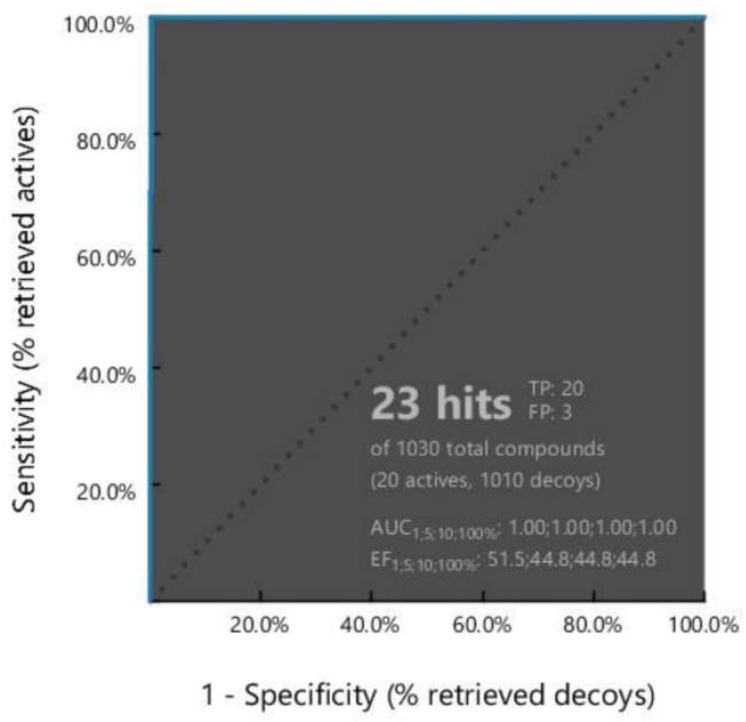
Receiver operating characteristic (ROC) curve generated based on the recognize ability of the active to decoy compounds of the structure-based pharmacophore model. The pharmacophore model was validated using a set of 20 FAK1 active and 1010 decoy compounds.

A validated pharmacophore analysis is necessary for ensuring the quality of the molecular model and determining the authenticity of the pharmacophore analysis. A structure-based pharmacophore model generated in this study was tested before database screening to determine if the models are capable of discriminating between active compounds and decoys. Validation of the pharmacophore model was carried out using 20 known antagonists of FAK1 (Table S1), and corresponding 1010 decoy compounds (Supplementary File S1) obtained from the enhanced Database of Useful Decoys (DUDe). As a first step to validate the model, the active test set with inhibitor constant IC_50_ values was merged with the decoy compounds, and an initial screening was conducted. An analysis of the receiver operating characteristic curve (ROC) measured the performance of the classification model, such as the AUC and EF values of the compounds. AUC provides a summary of the model’s performance and is used to express the degree of separability using ROC graphs. ROC graphs express the performance of classification models. Models with a higher AUC value should be more predictable. As the AUC value ranges from 0 to 1, the model with 100% accuracy has a value of 1. In the validation process, the early enrichment factor (EF1%) was 51.5 with an excellent AUC (area under the ROC curve) value in the 0.1% threshold, which was 100.00, which proved that our model has ability to distinguish true actives from decoy compounds (Figure 2).

### 2.3. Dataset Generation for Pharmacophore-Base Screening

The generation of a database is one of the most important steps in the screening process for finding the best lead molecule. The ZINC database is a collection of commercially available chemical compounds that provides information about the molecule’s molecular weight, chemical structure, physical and chemical properties against biologically active macromolecules. It contains over 230 million purchasable compounds in 3D format on a freely accessible website. A purchasable compound database library provides information about various compounds from different vendors, such as Ambinter. ZINCPharmer was used to generate the database for pharmacophore-based virtual screening using previously generated pharmacophore models. The ZINC database consists of millions of drug-like, purchasable products and FDA-approved drugs. It was initially searched for hits in the database on ZINC purchasable products and ZINC natural derivatives. A maximum of 0.5 Å RMSD was used as input parameters for ZINCPharmer, and a total of 235,000 compounds were retrieved for further screening. The database of hit compounds from ZINCPharmer was downloaded and saved for further screening.

### 2.4. Pharmacophore-Based Virtual Screening

A pharmacophore interaction feature derived from protein–ligand interactions was applied to 230,000 natural compounds. In the screening process, relative pharmacophore was used as a scoring function, including all query features as screening modes and omitting a maximum of four (4) features. To increase the pharmacophore fit score, some features were omitted during the screening process, as it is difficult to match all query features. A higher score indicates that the compound will have better activity against the targeted macromolecules when adapted to the desired environment. A total of twenty-three hits were generated with fit scores ranging from 88.20 to 125.02 that matched all pharmacophore features. It is usually the geometric fit of features to the pharmacophore model based on a 3D structure that is shown as a pharmacophore fit value. The most highly suited molecule to the validated pharmacophore model should exhibit activity against the FAK1 target protein. Compounds that were listed as hits were retrieved and saved for further evaluation.

### 2.5. Binding Site Identification and Receptor Grid Generation

**Figure 3:**
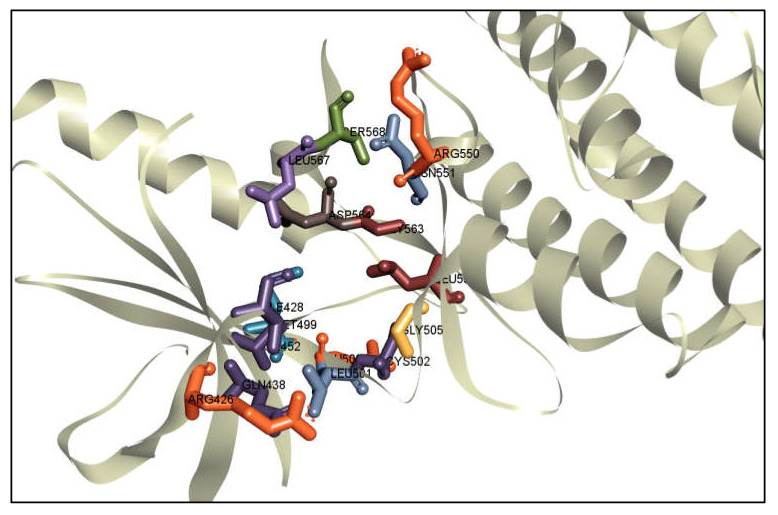
Showing the active site and correspondence binding site of FAK1 protein. Cylinder shapes with red, purple, orange, yellow, blue colors and so on, respectively, with their binding site position of the FAK1 protein.

The combined binding position of the active site (AS) was determined by identifying the AS of the FAK1 using the CASTpi server. A protein’s active pocket was analyzed to identify the binding site residue. Active site pocket analysis showed binding site locations at residues ARG550, ASN557, SER558, ASP564, LEU567, GLY563, LEU553, MET499, GLU500, ALA452, LEU501, GLN438 and ARG426. These positions are shown as cylinder shapes in various colors, including with red, green, pink, blue, green, and so on (Figure 3). The server-identified binding sites were used to generate a receptor grid during the molecular docking simulation procedure. The binding sites identified by the server were utilized to generate a receptor grid during the molecular docking simulation process with grid box dimensions of X = 98.39, Y = 102.46 and Z = 69.21 in angstrom (Å).

### 2.6. Molecular Docking

The binding affinity of the hit compounds to the target FAK1 protein using molecular docking, a crucial stage in drug development, was evaluated. PyRx tools Autodock Vina were used to dock specific compounds with FAK1 in order to assess their binding affinity, which meets the pharmacophore model’s assumptions. A total of four compounds, Pubchem CID24601203, CID1893370, CID16355541 and CID16467343 with binding affinities −10.4 kcal/mol, −10.1 kcal/mol, −9.7 kcal/mol, and −9.5 kcal/mol (Table 2), were found to have better binding affinity than the FAK1 antagonist Pubchem CID58522531 (−9 kcal/mol), which was used in the main pharmacophore model generation. There are differences in the binding affinity for all hit substances in Table S2. Interestingly, compounds with higher docking scores were suggested as having better binding affinity to the target protein. 

### 2.7. Interpretation of Protein-Ligands Interactions

**Figure 4:**
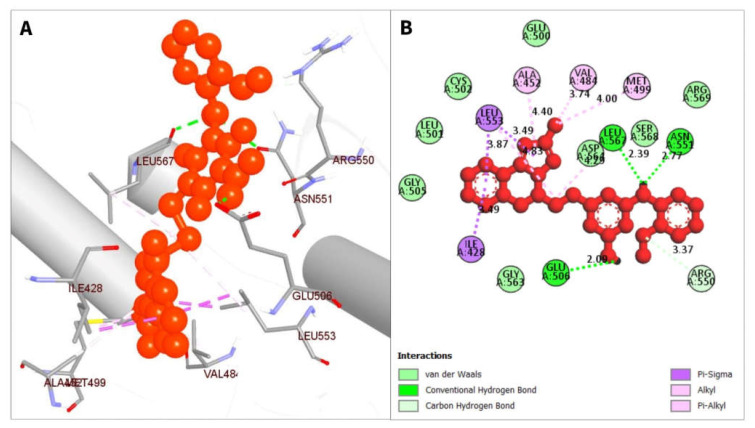
The non-bonded interaction of the CID24601203 and main protease from FAK1 at certain simulation times. Herein, (**A**) representing the 3D protein-ligand interaction and (**B**) representing 2D interaction of the protein with the ligand.

The non-bonded interactions between the ligand molecules and the FAK1 protein are shown in Figure 4. The compound CID24601203 and protein complex was stabilized by three hydrogen bonds at ASN551, LEU567, and GLU506, four alkyl bonds at LEU553, LEU567, VAL484, and MET499, two pi-sigma bonds at ILE428 and LEU553, one carbon hydrogen bond at ARG550, and one pi-alkyl bond at ALA452 (Table 3).

**Figure 5:**
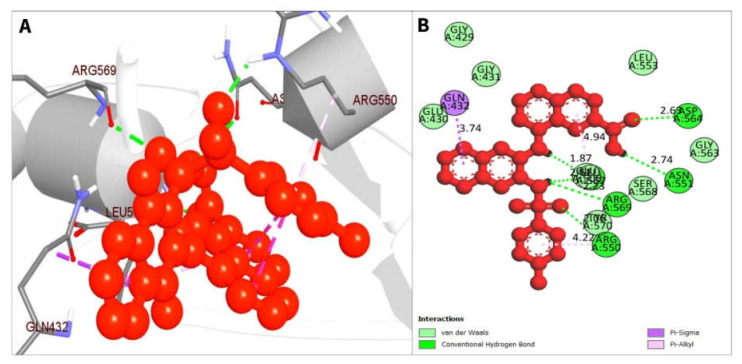
The non-bonded interaction of the CID1893370 and main protease from FAK1 at certain simulation times. Herein, (**A**) representing the 3D protein-ligand interaction and (**B**) representing 2D interaction of the protein with the ligand.

The compound CID1893370 and FAK1 protein complex formed six conventional hydrogen bond bonds at ARG550, ASP564, LEU567, ASN551, LEU567, and ARG569 position. The Pi-Sigma Pi-Alkyl bonds at the positions of GLN432, LEU567 and ARG550, respectively (Figure 5; Table 3).

**Figure 6:**
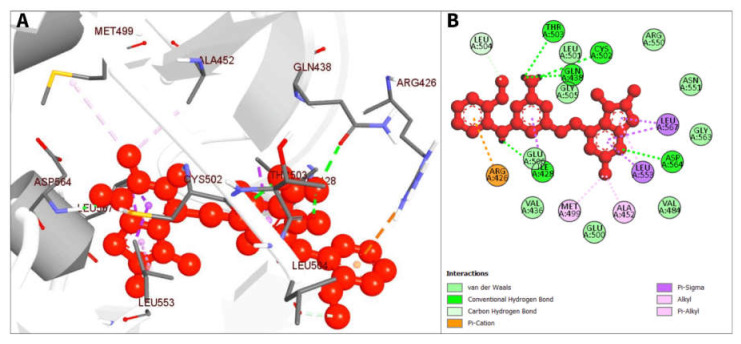
The non-bonded interaction of the CID16355541 and main protease from FAK1 at certain simulation times. Herein, (**A**) representing the 3D protein-ligand interaction and (**B**) representing 2D interaction of the protein with the ligand.

The compound CID16355541 and FAK1 protein complex created five conventional hydrogen bonds at ASP564, ILE428, GLN438, THR503 and CYS502, one carbon hydrogen bond at LEU504, one Pi-Cation bond at ARG426, four Pi-Sigma bonds at ILE428, LEU553, LEU567, and LEU567, two alkyl bonds at ALA452 and MET499 and one Pi-Alkyl bond at LEU553. Binding to the active sites may lead to the possible inhibition of the target molecule (Figure 6; Table 3).

**Figure 7:**
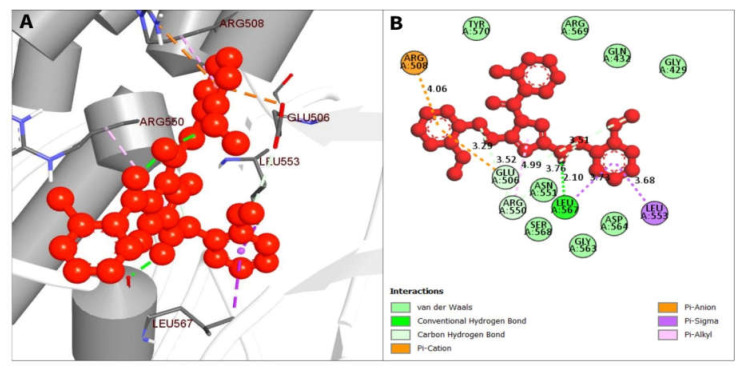
The non-bonded interaction of the CID16467343 and main protease from FAK1 at certain simulation times. Herein, (**A**) representing the 3D protein-ligand interaction and (**B**) representing 2D interaction of the protein with the ligand.

The compound CID16467343 and FAK1 protein complex formed three conventional hydrogen bond bonds at LEU567, and three carbon hydrogen bonds at ARG550, ARG550 and GLU506 position. The Pi-Cation, Pi-Anion and Pi-Sigma bond the positions of ARG508, GLU506, LEU553, and LEU567, respectively (Figure 7; Table 3). 

### 2.8. Absorption, Distribution, Metabolism and Excretion (ADME) and Toxicity Test Analysis 

#### 2.8.1. ADME Properties Analysis

The analysis of pharmakon (drug) and kinetikos (movement), combinedly known as pharmacokinetics (PK) properties analysis, is a crucial step in the process of developing new drugs. However, it focuses primarily on the ADME properties and incorporates physiochemical traits, such as lipophilicity, water solubility, pharmacokinetics, medication likeness, and medicinal chemistry. It also offers a potential hypothesis for choosing the best drug candidates. Pharmacophore analysis can reveal a compound’s xenobiotic regulation characteristics prior to entering it into the preclinical testing. The pharmacophore properties of the three drug-like compounds were ascertained using the SwissADME server. Lipophilicity is a property of drug-like compounds that allows them to dissolve in fats, oils, and nonpolar solvents. A compound’s lipophilicity means it can easily diffuse through the cell membrane; hence, oral preparation is not appropriate. Additionally, an injectable dosage form may be an effective method to achieve the rapid onset of action since gastrointestinal absorption is low. The table illustrates that the result of three compounds, such as CID2460123, CID1893370 and CID16355541, fitted more as a druggable compound except one compound, CID16467343. The medicinal chemistry data also show a similar result. Finally, pharmacophore properties indicate that the three compounds can be effective and druggable in the study (Table 4).

#### 2.8.2. Toxicity Analysis

In-silico toxicity measurement is a crucial procedure before clinical trials are begun for the selection of better lead compounds. Computer-based toxicity measurements have become popular due to their accuracy, rapidity, and accessibility, which enable them to provide information about any synthetic or natural compound. To identify the toxicity and adverse effects of the four selected compounds, we used both the free TEST tool and the ProTox II server. Several toxicological parameters were evaluated by the various software packages, including acute toxicity, hepatotoxicity, cytotoxicity, carcinogenicity, mutagenicity, and immunotoxicity, and a median lethal dose (LD50) in mg/kg was calculated based on weight. According to the ProTox-II server compound, Pubchem CID24601203, CID1893370, CID16355541 and CID16467343 belonged to class 4, and the LD50 range was also compiled (Table 5). 

### 2.9. MD Simulation

MD simulation evaluation molecular dynamics (MD) simulations were used to examine and evaluate the binding stability of protein-ligand complexes. Throughout the orientation time, the MD simulation recorded data focusing on intermolecular interaction. The stability of the protein–ligand complexes was determined using a 100 ns MD simulation. The root mean square deviation (RMSD), root mean square fluctuation (RMSF), intramolecular hydrogen bonding (Intra HB), and protein–ligand interaction analyses are used to present the MD simulation results (P–L contact).

#### 2.9.1. RMSD Analysis

**Figure 8:**
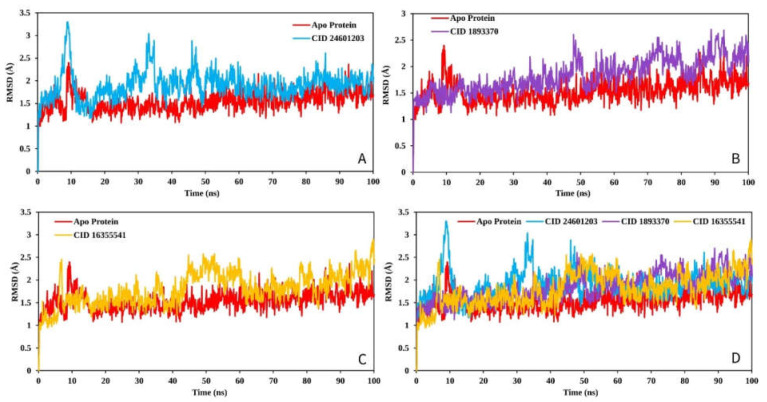
The RMSD values extracted for the Cα atoms of the three selected compounds in complex with the FAK1 protein (red) for the compounds (**A**) CID: 24601203 (blue), (**B**) CID: 1893370 (purple), (**C**) CID: 16355541 (yellow) and, (**D**) all the RMSD for all compounds and the protein together.

The RMSD is used to quantify the average change in position of a chosen set of atoms relative to a reference atom. The RMSD analysis is used to describe the system equilibration in terms of stability and reliability. The smaller range of RMSD and constant fluctuation throughout the simulation imply that the protein backbone is stable. On the other hand, a larger RMSD and/or significant variation from the native structure suggest that the protein–ligand combination is more unstable. The mean or average value change between a specific frame and a reference frame with a range order of 1–3 Å is entirely permissible, where a value larger than the required range indicates that the protein has undergone a significant conformational shift. The MD simulation with a time step of 100 ns was used to provide the RMSD that was calculated from RMSD (Figure 8).

#### 2.9.2. RMSF Analysis

**Figure 9:**
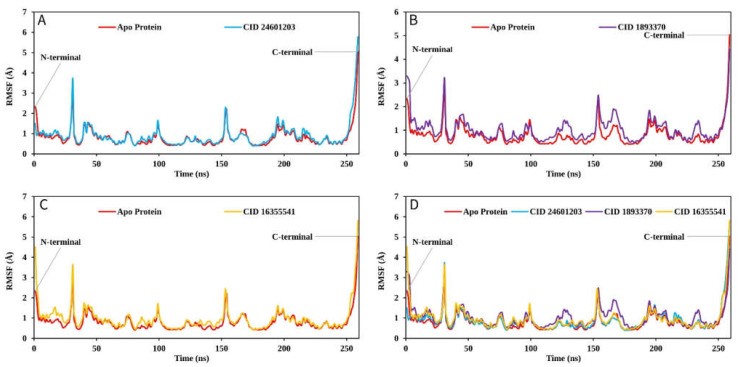
The RMSF values extracted for the Cα atoms of the five selected compounds in complex with the FAK1 protein (red) in complex with the compounds (**A**) CID: 24601203 (blue), (**B**) CID: 1893370 (purple), (**C**) CID: 16355541 (yellow) and (**D**) shows the RMSF for all compounds and the protein together.

The largest change in the case of the Apo protein was recorded between residue positions 30 and 40 aa, with a fluctuation of 3.81. CID:24601203 appeared to have the smallest average RMSF range between 0.5 and 1.1, as well as the lowest fluctuation between 150 and 158 aa when compared to the Apo protein structure (Figure 9A). However, the RMSF graph revealed that the FAK1 protein in association with CID: 1893370 (1.02 to 1.6) and CID: 16355541 (0.5–2.3) had average low and significant values when compared to the reference apo structure, as shown in Figure 9D. As previously stated, a low RMSF value suggests higher protein stability, and the RMSF values found in this study for each protein–ligand system were lower than those discovered for Apo protein.

#### 2.9.3. Protein–Ligand Contacts 

**Figure 10:**
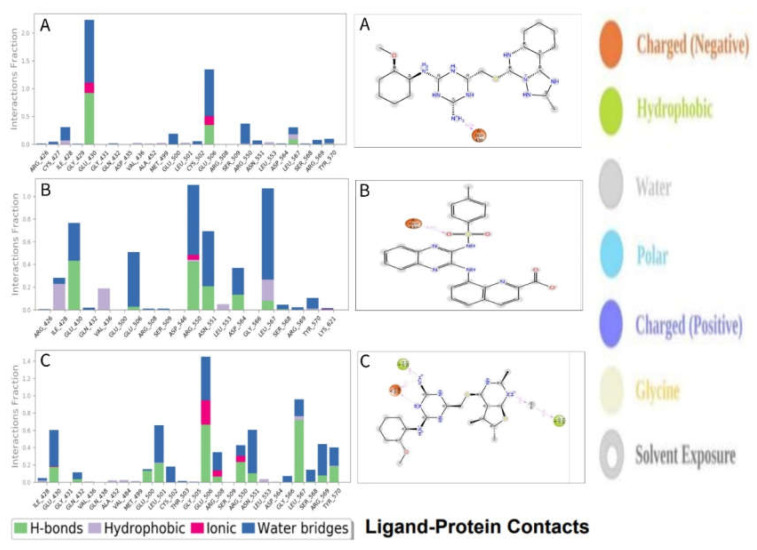
Schematic representation of interactions of selected ligand atoms with FAK1protein residues shown for interactions that occur more than 30.0% of the simulation time between the protein and the compounds (**A**) CID: 24601203, (**B**) CID: 1893370, and (**C**) CID: 16355541 in the selected trajectory (0.00 through 100.00 ns).

The bonding relationship between the compounds and the target protein is crucial in terms of stability and PK characteristics. For example, molecular hydrogen bonding influences drug selectivity, adsorption, and metabolism. As a result, a simulation interaction diagram was used to study the protein complex with the selected ligands and their intermolecular interactions (SID). Figure 10 depicts the interactions that occur for more than 30.0% of the simulation duration between the natural compound (CID: 24601203, CID: 1893370, and CID: 16355541) atoms and the FAK1 protein residues and are characterized by hydrogen, hydrophobic, ionic, and water bridge linkages. Figure 10 also includes a stacked bar chart representation of the protein–ligand interactions discovered during the 100 ns simulation run. An interaction fraction value (IFV) can be used to describe the interaction between the protein and the ligands. For example, an IFV value of 0.7 indicates that the specific interaction is maintained for 70% of the simulation duration. Because some protein residues may make several interactions of the same subtype with the ligand, values greater than 1.0 (>100%) are feasible. The ARG side chain, for example, has four H-bond donors that can form four hydrogen bond interactions with a single H-bond acceptor.

#### 2.9.4. MM-GBSA Analysis 

**Figure 11:**
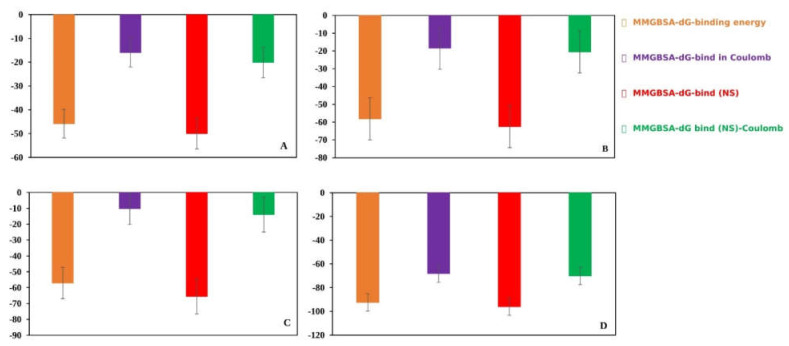
Different energy components and net MM-GBSA binding free energy (kcal/mol) along with the standard deviation values calculated from 100 ns MD simulation trajectory of FAK1 protein in complex with the selected compounds (**A**) CID: 24601203, (**B**) CID: 1893370, and (**C**) CID: 16355541 and (**D**) control.

The MM-GBSA approach helps to determine the binding free energy of a molecule to the target protein. The binding free energy of the selected molecules to the target protein were evaluated based on the MD simulation trajectory and are represented in Figure 11. The MM-GBSA of the complex structure was computed for every single frame generated for the 100 ns MD simulation trajectory. The analysis of the complex structure identified higher net negative binding free energy values of −45.8499 ± 6.03, −58.1706 ± 11.35, −57.0858 ± 9.89, and −92.4586 ± 4.86 kcal/mol for the three selected molecules (A) CID: 24601203, (B) CID: 1893370, and (C) CID: 16355541 and control, respectively, with the target protein (Figure 11).

## 3. Discussion

Pancreatic adenocarcinoma is a deadly illness that is expected to become the second biggest cause of cancer mortality globally [1]. The treatment of pancreatic adenocarcinoma is developing, with the advent of new surgical procedures and pharmacological therapies such as laparoscopic techniques and neoadjuvant chemoradiotherapy; however, this has resulted in only minor improvements in patient outcomes [20]. Given these considerations, new and more effective anti-PANC chemotherapeutics are urgently required. Therefore, we utilized a ligand-based drug design (LBDD) for the identification of purchasable substrate analogs that interact with the target molecule resulting in desired pharmacological effects against PANC. The main objective of LBDD methods is to identify known ligands for a target and establish a structure–activity relationship (SAR) between their physiochemical characteristics and drugs activities. This information can be used to improve existing medications or to help create new medications with increased activity.

Computer-aided drug design (CADD) ushers in a new era of medicine by making processes more cost-effective, saving time, and reducing labor costs. This makes drug discovery more feasible. To discover drug candidates with the best biological effectiveness, procedures such as virtual screening, molecular docking, and ADMET are utilized. Understanding the illness’s mechanism, finding the disease-associated protein, and devising a ligand-binding method for the protein may all contribute to a reduction in disease severity. CADD helps identify particular target molecules based on their behavior and mode of ligand binding. Molecular docking, on the other hand, identifies the most prevalent binding modes between a ligand and a protein. Small molecule candidates may therefore be identified as potential therapeutic agents for a certain disease. 

In this study, ligand-based pharmacophore drug design process was utilized includes pharmacophore modeling and validation; pharmacophore based virtual screening, virtual hits profiling and lead identification. The comprehensive drug design approach was used to screen purchasable compounds library for their ability to treat pancreatic cancer. The four best compounds have been chosen from the compound’s library with the greatest binding affinity based on their molecular docking score. The highest bonding was documented to compounds Pubchem CID24601203, CID1893370, CID16355541 and CID16467343 with binding scores of −10.4 kcal/mol, −10.1 kcal/mol, −9.7 kcal/mol, and −9.5 kcal/mol, respectively. It is a very good value and show the characteristic feature of the purchasable compound to be treated as a drug. The drug-like qualities of the selected compounds were proven using Lipinski’s rule of five (RO5) [21]. Analyses of ADME approaches were used to study the metabolite kinetics in small molecular candidates. The best GI absorption of the chosen compounds without blood–brain barrier (BBB) permeation was found through pharmacokinetic analysis. The candidate molecule has a consensus log Po/w value of 5, which denotes that it is lipophilic and can thus cross lipid membranes found inside the body. The chosen compounds were found to have good drug-like properties according to Lipinski’s rule of five (LR5), which evaluates the drug-like properties of selected compounds to determine whether they are orally bioavailable for humans. Four substances were evaluated for their pharmacokinetic (PK) characteristics, and each was determined to be of substantial value. The study predicted the possible binding to toxicity targets using a collection of protein–ligand-based pharmacophores. Toxicity targets are protein targets which are associated with adverse drug reactions and toxic effects. Several toxicological parameters were evaluated by the various software packages, including acute toxicity, hepatotoxicity, cytotoxicity, carcinogenicity, mutagenicity, and immunotoxicity, and a median lethal dose (LD50) in mg/kg was calculated based on weight. The findings revealed that the four chosen compounds were non-toxic or low toxic, belonging to toxicity class 4, and that their LD50 values were within the range.

## 4. Materials and Methods

### 4.1. Ligand-Based Pharmacophore Modeling and Virtual Screening

#### 4.1.1. Ligand-Based Pharmacophore Modeling

An extensive literature search along with a review of all ChEMBL target annotations (based on high-confidence activity data) led to the generation of active antagonists of focal adhesion kinase 1 protein (FAK1). To generate ligand-based pharmacophore models, the 20 active antagonists (Table 1) derived from ChEMBL (https://www.ebi.ac.uk/chembl/ (accessed on 7 May 2020)) and literature search were docked by PyRx AutoDock Vina using the scoring functions for the FAK1 protein (PDB ID: 3BZ3). The best compound with highest binding affinity (kcal/mol) was selected for ligand-based pharmacophore modeling. The best scoring compound, in complex with FAK1, was used to interact with purchasable compounds and retrieve hits. A ligand-based pharmacophore model was generated using LigandScout 4.3 advance software. The advanced software makes the interaction between inhibitors and critical amino acids in the target proteins. In addition to interpreting ligand–receptor interactions, the software takes into account different pharmacophore features, including hydrogen bond donor and acceptor regions, hydrophilic and hydrophobic regions, and hydrogen bond acceptors [22]. A stepwise algorithm was used to detect the number of aromatic rings, the state of hybridization, the pattern of binding, as well as the distance between receptor molecules. The ligand scout was used to identify better and optimal compound structures by removing hydrophilic properties from the protein and adding or removing features to the active site necessary to maintain sterically circumference.

#### 4.1.2. Pharmacophore Model Validation

A pharmacophore validated by a protein-ligand interaction generally helps to determine whether a compound is active or inactive. The ligand-based pharmacophore model generated from the protein-ligand complex was tested by screening a set of 20 known active substances and correspondence with 1010 decoy compounds obtained from the DUD-E database of decoys. In LigandScout 4.3, the “create screening database” option was used to convert DUD-E to .ldb format before screening [23]. The GH score and early enrichment factor (EF) were used to assess the quality of the ligand-based model.

#### 4.1.3. Dataset Generation for Pharmacophore-Base Screening

Based on the pharmacophore model, the structurally novel and active molecules can be identified by the completion of virtual screening. Chemical databases such as Zinc database (https://zinc.docking.org/, accessed on 7 May 2020) can be used to identify possible lead compounds [24]. Compounds from the database can be searched using the structural information, name of the compound, or chemical smile ID. Analyses were conducted of the physical and chemical properties, such as 2D and 3D structure calculations, boiling point and melting point. A compound’s molecular weight, crystal structure, and biological application can also be determined [25]. In the case of a desired compound, the compound having the most similar features was given top priority. The compound had to match the required pharmacopeial features and interact with our target protein. The potential hits were selected by matching their maximum features to the query pharmacophore. Initially, the project screened a ZINC purchasable product library using the ZINCPharmer program (http://zincpharmer.csb.pitt.edu/pharmer.html, accessed on 7 May 2020) server for target XIAP based on pharmacophore features.

#### 4.1.4. Pharmacophore-Based Virtual Screening

ZINCPharmer generated a database that was screened against the validated ligand-based pharmacophores. The new version of LigandScout 4.3 helps create and obtain a 3D model of protein–ligand interactions as well as the ability to change compounds into the specific file format (iDB). These compounds were passed directly into the database list for quick virtual screening based on pharmacophore features [26]. Several system features were omitted by selecting relative pharmacophore fits as a method of securitizing. A pharmacophore fit score was used to organize compounds that fit the pharmacophore, and they were then subjected to further validation [27].

### 4.2. Molecular Docking Based Virtual Screening

In computational biology, protein preparation refers to the conversion of macromolecular structures into a form that is more suitable for computation. In the process of docking crystal structures of proteins, it is necessary to prepare the structures for other purposes not included in the X-ray crystal structure refinement process, such as adjusting hydrogen bonds, removing atomic collisions, and performing other operations. This study used the protein data bank to obtain the desired 3D structure of FAK1 protein (PDB ID: 3BZ3). The structure was determined experimentally and validated using the X-ray diffraction method with a resolution of 2.20 Å and R-value free score of 0.234 which is significantly less than the standard value of 0.16. The X-ray crystallography structure of our desire protein was prepared by removing water, metal ions and cofactors, adding polar hydrogen bonds, merging non-polar hydrogen bonds, and calculating gasteiger charges using AutoDockTools (ADT) [28]. The energy and bond angles of selected hit compounds were optimized by default using the universal force field (UFF) for each of the ligands.

#### 4.2.1. Active Site Identification and Grid Generation

To treat a particular disease, the ligand or drug molecule has to bind to a specific site on protein. The improper attachment of the ligand may result in several side effects in the body as well as a higher possibility of toxicities. The binding affinities of zinc compounds depend on several features, including H bond donors, hydrophobic or hydrophilic interactions, ionization, and chelation. The binding site of our desired protein was found using BIOVA Discovery Studio Visualizer Tool 16.1.0. Furthermore, PrankWeb (https://prankweb.cz/ (accessed on 27 October 2022)) server was used to analyze the probable binding sites of the desired protein structure. An algorithm based on machine learning was used by the server to predict ligand binding sites from protein structure. The PyRx software was used to generate the receptor grid after selecting the active site of the protein [29].

#### 4.2.2. Molecular Docking

After selecting the active site of the protein, the receptor grid was generated using PyRx software. The PyRx virtual screening software was used to perform molecular docking on selected hits from pharmacophore screening. PyRx is a virtual screening software used in computational biology to identify potential drug candidates [30]. The Lamarckian genetic algorithm (LGA) is a scoring function included in AutoDock and AutoDock Vina. In this study, molecular docking interactions were performed using the PyRx tool AutoDock Vina. BIOVA Discovery Studio Visualizer Tool 16.1.0 was used to retrieve and visualize the docked compound with better binding affinity (kcal/mol).

### 4.3. Absorption, Distribution, Metabolism and Excretion (ADME) and Toxicity Test

#### 4.3.1. Absorption, Distribution, Metabolism and Excretion (ADME)

The ADME properties of a molecule are some of the main criteria before its development into a drug. For the early stage of prediction, computer-based prediction is essential, as many drug candidates cannot fit the clinical trial demand [31]. ADME profiles directly affect physiochemical properties, hydrophobicity, lipophilicity, the gastrointestinal environment, and the blood brain barrier before drugs are excreted by the body through urine and feces. The Swiss-ADME (http://www.swissadme.ch/ (accessed on 27 October 2022)) server was used to evaluate ADME properties, such as solubility, GIT absorption, and bioavailability [32].

#### 4.3.2. Toxicity Test

Compounds can be evaluated for their safety profile using computational-based methods for evaluating toxicity. Toxicology profile can be used to evaluate and determine mutagenicity, carcinogenicity, LD50 value, and immunotoxicity quantitatively and qualitatively. A free software tool called TEST (Toxicity Estimation Software Tool) was used in this study to estimate toxicity of our compounds without requiring any external software. Furthermore, a toxicity estimator TEST tool was used for selective molecules using quantitative structure–activity relationship (QSAR) methods. To determine the toxic effect of the selected four compounds, ProTox-II (http://tox.charite.de/protox_II (accessed on 27 October 2022)) server was used [33]. There are different toxicological pathways provided by this website, such as nuclear-receptor-signaling pathways and stress-response pathways. 

### 4.4. Molecular Dynamics (MD) Simulation

To further evaluate the binding mode of our candidate molecules, the best poses obtained from re-docking studies were tested through 100 ns molecular dynamics simulations. MD simulations were performed under Linux program with the Desmond module in Schrödinger Release 2020-3 [34]. As a first step, a simple point charge (SPC) water model with orthorhombic box boundary conditions was used to solve the complex protein–ligand interaction. There is a buffer box calculation method with the box distances 15 Å (a = 5 Å, b = 5 Å, and c = 5 Å) on both sides for all the complex’s atoms. In order to neutralize the system, Na+ and Cl^−^ were added at a salt concentration of 0.15 M. The MD simulation was conducted at constant pressure (1.01325 bar) and temperature (300 K) with recoding intervals of 50 ps energy using the OPLS-2005 force field.

#### Simulation Trajectory Analysis

The quality of the MD simulation was confirmed, and the simulation event was examined by using the simulation interaction diagram (SID), which is included in the Schrödinger program. The RMSD, RMSF, protein–ligand interactions (P-L contacts), and hydrogen bond interactions identified in the trajectory were used to determine the stability of the complex structure.

### 4.5. RMSD Analysis 

The RMSD of a protein–ligand complex system calculates the average distance brought on by the dislocation of a particular atom over a predetermined amount of time and denotes the stability of the protein. Following the initial alignment of the reference frame backbone and protein structures, the RMSD of the entire system was estimated over nearly the same length of time as the MD simulations (in our instance, 100 ns).

### 4.6. RMSF Analysis

Besides estimating the average observed atomic changes in relation to the number of atoms, the resulting RMSF information is useful in determining the local conformational change of a protein linked with ligands.

### 4.7. MM-GBSA Analysis

The MM-GBSA approach is frequently used to determine the binding free energy of a chemical compound with a protein or a free ligand. A complicated system’s MM-GBSA can be calculated using the MD simulation trajectory, which is more precise than the majority of scoring functions. Therefore, the Prime MM-GBSA module in the Schrödinger Maestro package was used to apply the MM-GBSA methods in order to calculate the binding free energy (Gbind) of the selected compounds in the complex with the FAK1 protein.

## 5. Conclusions

Focal adhesion kinase 1 (FAK1) protein is responsible for the growth-factor signaling, cell proliferation, cell survival and migration that lead to triggering of pancreatic cancer. However, no effective drug has been identified to inhibit the FAK1 protein against pancreatic cancer. Therefore, this study aimed to find a purchasable and effective compound that might block the function of protein and impede cancer growth. As a result, the research applied a broad range of computational methods, including the ligand-based pharmacophore model, molecular docking, ADMET, MD simulation, and MM-GBSA approaches, and identified three promising therapeutic candidates such as Pubchem CID24601203, CID1893370, and CID16355541, with binding scores of −10.4, −10.1, and −9.7 kcal/mol, respectively. However, these purchasable compounds must be subjected to in vitro and in vivo investigations to evaluate their effectiveness and safety as anti-FAK1 drugs in humans. It is both therapeutically and commercially feasible to develop the selected compounds as drug candidates against FACK1.

## Figures and Tables

**Table 1:** List of 10 known active antagonists of FAK1 protein and their binding affinity toward the protein generated through molecular docking method.

PubChem ID	IC50 (nM)	Chemical Name	Chemical Formula	Chemical Structure	Binding Affinity
58522531	6	BDBM134122	C_27_H_25_F_4_N_5_O_5_	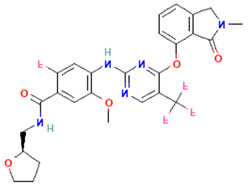	−9.0
58522578	1	BDBM134151	C_27_H_25_F_4_N_5_O_5_	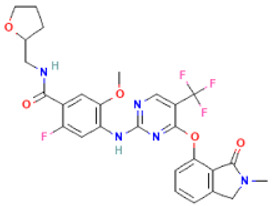	−8.7
58522559	1	BDBM134145	C_28_H_30_ClFN_6_O_4_	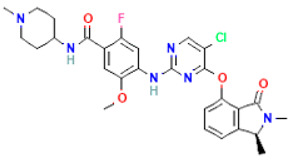	−8.4
58522525	1	BDBM134129	C_27_H_27_Cl_2_FN_6_O_4_	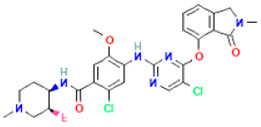	−8.0
58522543	6	BDBM134017	C_28_H_27_ClF_3_N_5_O_4_	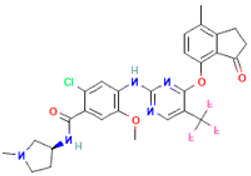	−8.0
58522647	1	BDBM134167	C_29_H_33_ClN_6_O_5_	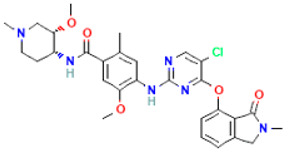	−7.9
58522593	4	BDBM134002	C_27_H_25_F_4_N_5_O_5_	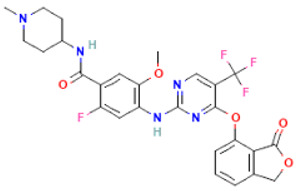	−7.7
58522523	4	BDBM134035	C_30_H_31_F_5_N_6_O_4_	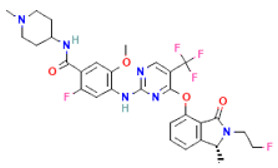	−7.6
58522553	1	BDBM134115	C_28_H_29_F_3_N_6_O_5_	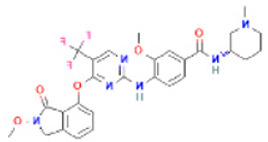	−7.5
58522562	1	BDBM134134	C_29_H_31_F_3_N_6_O_4_	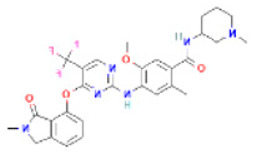	−7.5

**Table 2:** Docking score with FAK1 protein, docking score and molecular weight of the top four selected compounds.

Pubchem ID	Compound Name	Molecular Formula	Molecular Weight	Chemical Structure	Docking Score
24601203	ZINC13230575	C_21_H_19_N_9_OS	445.5	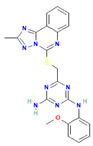	−10.4
1893370	SCHEMBL1790903	C_25_H_19_N_5_O_4_S	485.5	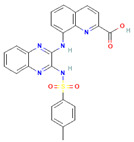	−10.1
16355541	AKOS033282660	C_20_H_21_N_7_OS_2_	439.6	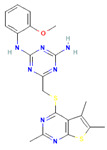	−9.7
16467343	AKOS005409633	C_25_H_24_ClN_5_O_3_	477.9	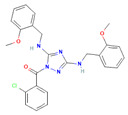	−9.5

**Table 3:** List of bonding interactions between selected four compounds with FAK1 protein.

PubChem CID	Residue	Distance	Category	Type
CID 24601203	ASN551	2.77	Hydrogen Bond	Conventional Hydrogen Bond
	LEU567	2.39	Hydrogen Bond	Conventional Hydrogen Bond
	GLU506	2.09	Hydrogen Bond	Conventional Hydrogen Bond
	ARG550	3.37	Hydrophobic	Carbon Hydrogen Bond
	ILE428	3.49	Hydrophobic	Pi-Sigma
	LEU553	3.89	Hydrophobic	Pi-Sigma
	LEU553	4.83	Hydrophobic	Alkyl
	LEU567	4.29	Hydrophobic	Alkyl
	VAL484	3.74	Hydrophobic	Alkyl
	MET499	3.99	Hydrophobic	Alkyl
	ALA452	4.39	Hydrophobic	Pi-Alkyl
CID 1893370	ARG550	2.05798	Hydrogen Bond	Conventional Hydrogen Bond
	ASP564	2.63012	Hydrogen Bond	Conventional Hydrogen Bond
	LEU567	1.869	Hydrogen Bond	Conventional Hydrogen Bond
	ASN551	2.73854	Hydrogen Bond	Conventional Hydrogen Bond
	LEU567	2.60894	Hydrogen Bond	Conventional Hydrogen Bond
	ARG569	2.23097	Hydrogen Bond	Conventional Hydrogen Bond
	GLN432	3.74021	Hydrophobic	Pi-Sigma
	LEU567	4.93986	Hydrophobic	Pi-Alkyl
	ARG550	4.22051	Hydrophobic	Pi-Alkyl
CID 16355541	ASP564	2.16221	Hydrogen Bond	Conventional Hydrogen Bond
	ILE428	2.76461	Hydrogen Bond	Conventional Hydrogen Bond
	GLN438	2.68776	Hydrogen Bond	Conventional Hydrogen Bond
	THR503	2.77995	Hydrogen Bond	Conventional Hydrogen Bond
	CYS502	2.79357	Hydrogen Bond	Conventional Hydrogen Bond
	LEU504	3.68955	Hydrogen Bond	Carbon Hydrogen Bond
	ARG426	3.99237	Electrostatic	Pi-Cation
	ILE428	3.62494	Hydrophobic	Pi-Sigma
	LEU553	3.33756	Hydrophobic	Pi-Sigma
	LEU567	3.706	Hydrophobic	Pi-Sigma
	LEU567	3.90901	Hydrophobic	Pi-Sigma
	ALA452	3.91212	Hydrophobic	Alkyl
	MET499	4.54299	Hydrophobic	Alkyl
	LEU553	4.57388	Hydrophobic	Pi-Alkyl
CID 16467343	LEU567	2.09727	Hydrogen Bond	Conventional Hydrogen Bond
	ARG550	3.52399	Hydrogen Bond	Carbon Hydrogen Bond
	ARG550	3.75615	Hydrogen Bond	Carbon Hydrogen Bond
	GLU506	3.51249	Hydrogen Bond	Carbon Hydrogen Bond
	ARG508	4.05546	Electrostatic	Pi-Cation
	GLU506	3.29313	Electrostatic	Pi-Anion
	LEU553	3.67551	Hydrophobic	Pi-Sigma
	LEU567	3.72557	Hydrophobic	Pi-Sigma

**Table 4:** List of pharmacokinetic properties (physico-chemical, lipophilicity, water solubility, drug likeness, and medicinal chemistry) of the selected four compounds.

Properties	Parameters	CID24601203	CID1893370	CID16355541	CID16467343
	MW (g/mol)	445.5 g/mol	485.5 g/mol	439.6 g/mol	477.9 g/mol
	Heavy atoms	32	35	30	34
	Arom. heavy atoms	25	26	21	23
	Rotatable bonds	4	6	6	10
	H-bond acceptors	6	7	6	5
	H-bond donors	7	3	2	2
	Molar Refractivity	123.29	133.42	122.35	131.77
Lipophilicity	Log Po/w	3.71	5.62	4.16	4.46
Water solubility	Log S (ESOL)	−5.15	−5.79	−5.68	−6.61
Pharmacokinetics	GI absorption	Low	Low	Low	High
Drug likeness	Lipinski, Violation	No	No	No	No
Medi. chemistry	Synth. accessibility	3.49	3.68	3.68	3.47

**Table 5:** List of toxicity properties (organ toxicity, toxicity endpoints, Tox21-Nuclear receptor signaling pathways, Tox21-Stress response pathway, Fathead minnow LC_50_ (96 h), developmental toxicity, oral rat LD_50_, bioaccumulation factor) of the selected four compounds.

Endpoint	Target	CID 24601203	CID 1893370	CID 16355541	CID 16467343
Organ toxicity	Hepatotoxicity	Inactive	Inactive	Inactive	Inactive
Toxicity endpoints	Carcinogenicity	Inactive	Inactive	Inactive	Inactive
	Immunotoxicity	Active	Light active	Light active	Inactive
	Mutagenicity	Inactive	Inactive	Inactive	Inactive
	Cytotoxicity	Inactive	Inactive	Inactive	Inactive
	Toxicity class	4	4	4	4
Tox21-Nuclear receptor signaling pathways	Androgen receptor (AR)	Inactive	Inactive	Active	Inactive
	Aryl hydrocarbon receptor (AhR)	Inactive	Inactive	Active	Inactive
Tox21-Stress response pathway	Heat shock factor response element	Inactive	Inactive	Active	Inactive
Fathead minnow LC50 (96 h)	(mg/L)	0.49	3.12 × 10^−2^	0.41	1.37 × 10^−2^
48-h Daphnia magna LC50	−Log10(mol/L)	4.54	4.6	4.33	2.68
Developmental toxicity	value	0.75	0.83	0.75	0.51
Oral rat LD50	mg/kg	600.89	1181.5	543.77	1102.13
Bioaccumulation factor	Log10	N/A	1.3	0.66	1.44

## Data Availability

Data is contained within the article and supplementary material.

## Supplementary Materials

The following supporting information can be downloaded at: https://www.mdpi.com/1424-8247/16/1/120/s1, Figure S1: Ten ligand-based model overview; Table S1: List of 20 known active antagonist of FAK1 protein and their binding affinity towards the protein generated through molecular docking method; Table S2: Generated ten ligand-based model and score. Table S3. List of MM/GBSA component and their energy with standard error value of the selected three compounds and Control.
