# Supplementary material for: Integrative Ligand-Based Pharmacophore Modeling, Virtual Screening, and Molecular Docking Simulation Approaches Identified Potential Lead Compounds against Pancreatic Cancer by Targeting FAK1
Source: Pharmaceuticals (Basel). 2023 Jan 13;16(1):120. doi: 10.3390/ph16010120 (PMC9912262; doi:10.3390/ph16010120)

Table S1. List of 20 known active antagonist of FAK1 protein and their binding affinity towards the protein generated through molecular docking method.

PubChem ID	IC50 (nM)	Chemical Name	Chemical Formula	Chemical Structure	Binding Affinity
58522531	6	BDBM134122	$C_{27}H_{25}F_4N_5O_5$	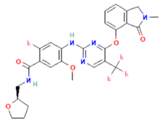	-9
58522578	1	BDBM134151	$C_{27}H_{25}F_4N_5O_5$	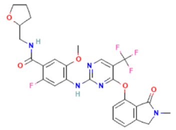	-8.7
58522559	1	BDBM134145	$C_{28}H_{30}ClFN_6O_4$	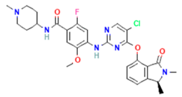	-8.4
58522525	1	BDBM134129	$C_{27}H_{27}Cl_2FN_6O_4$	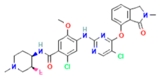	-8.0

58522543	6	BDBM134017	$C_{28}H_{27}ClF_3N_5O_4$	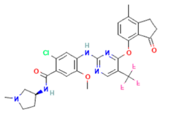	-8.0
58522647	1	BDBM134167	$C_{29}H_{33}ClN_6O_5$	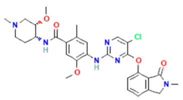	-7.9
58522593	4	BDBM134002	$C_{27}H_{25}F_4N_5O_5$	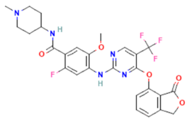	-7.7
58522523	4	BDBM134035	$C_{30}H_{31}F_5N_6O_4$	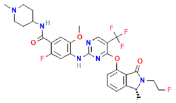	-7.6
58522553	1	BDBM134115	$C_{28}H_{29}F_3N_6O_5$	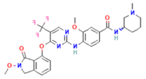	-7.5

58522562	1	BDBM134134	$C_{29}H_{31}F_{3}N_{6}O_{4}$	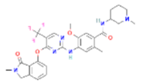	-7.5
58522601	2	BDBM134195	$C_{23}H_{24}ClN_{5}O_{3}$	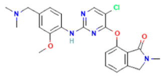	-7.4
58522569	3	BDBM134192	$C_{23}H_{20}F_{3}N_{5}O_{4}$	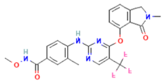	-7.2
11525210	4	BDBM50184049	C23H23N5O5	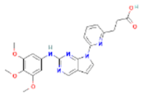	-7.1
58522632	8	BDBM134053	$C_{31}H_{34}F_{3}N_{5}O_{3}$	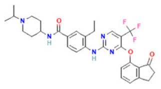	-7.1
44563062	0.94	BDBM50246286	$C_{21}H_{22}F_{3}N_{5}O_{4}S_{2}$	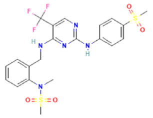	-7.0

46208089	2	BDBM134081	$C_{25}H_{26}ClN_5O_3$	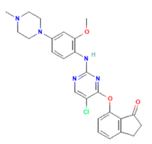	-7.8
46208482	1	BDBM134146	$C_{28}H_{30}ClFN_6O_4$	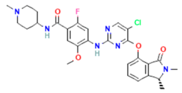	-6.8
46208388	8	BDBM126852	$C_{27}H_{28}F_3N_5O_5$	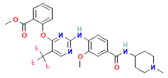	-6.6
58522590	2	BDBM134181	$C_{25}H_{24}F_3N_5O_4$	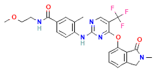	-6.5

Table S2: Generated ten ligand based model and score

Name	Score
Model 1	0.9180
Model 2	0.9170
Model 3	0.9157
Model 4	0.9108

Model 5	0.9095
Model 6	0.9075
Model 7	0.9038
Model 8	0.8612
Model 9	0.8606
Model 10	0.8596

Table S3: List of MM/GBSA component and their energy with standard error value of the selected three compounds and Control.

**Compound**	**MMGBSA-dG-binding energy**	**MMGBSA-dG-bind in Coulomb**	**MMGBSA-dG-bind(NS)**	**MMGBSA-dG bind(NS)-Coulomb**
CID24601203	−45.8499±6.03	−15.9768±6.03	−50.0216±6.42	−20.1245±6.42
CID1893370	−58.1706±11.35	−18.4305±11.35	−62.5148±11.45	−20.5074±11.45
CID16355541	−57.0858±9.89	−10.2166±9.89	−65.5522±10.99	−13.9453±10.99
Apo protein	−92.4586±4.86	−68.181±4.89	−96.0087±5.09	−70.1273±5.09

Figure S1 : Ten ligand based model overview

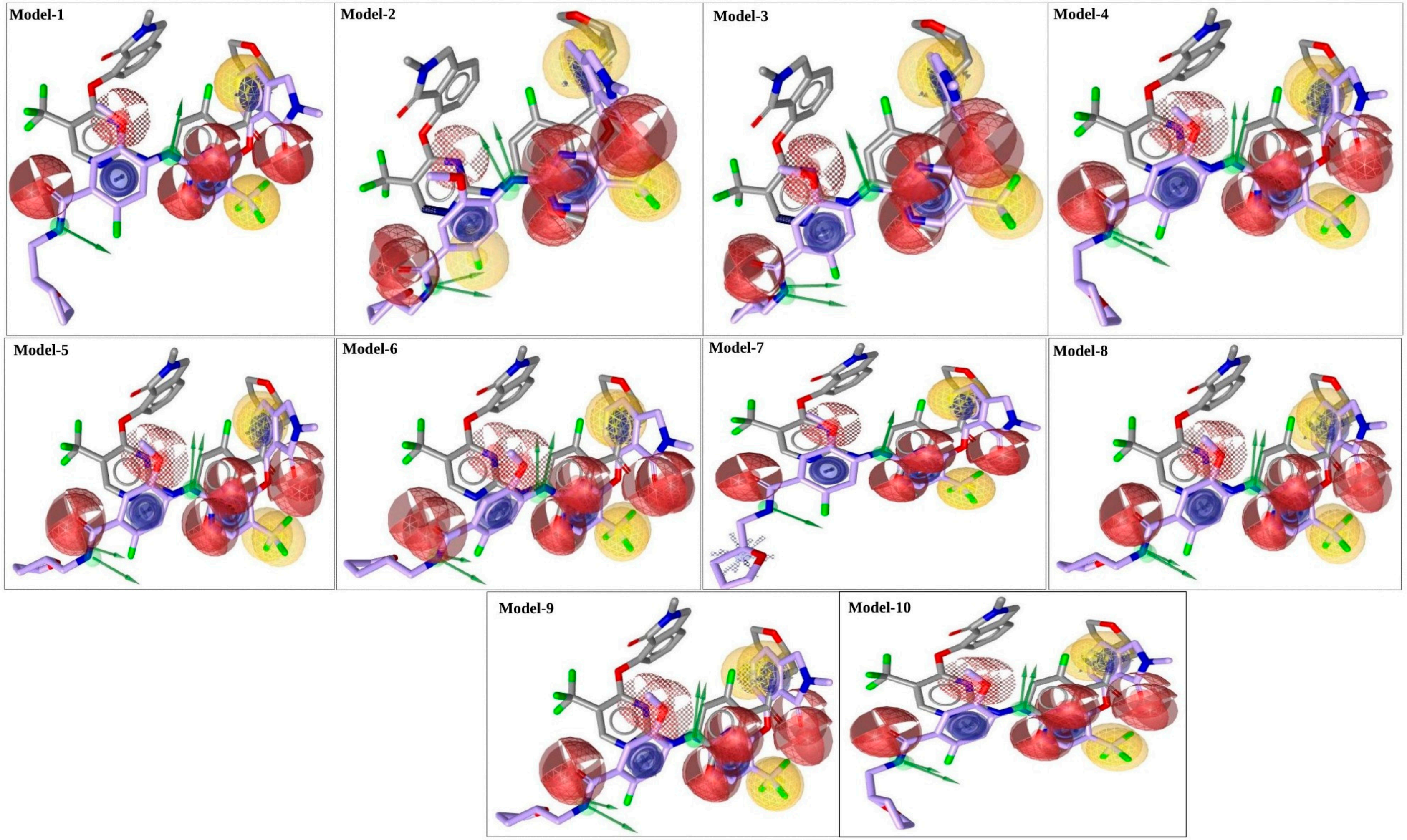

Supplement: Supplementary file 1 [file pharmaceuticals-16-00120-s001.zip › pharmaceuticals-2029558-Supplementary File.pdf]
